# Spatio-Temporal Variation in Landscape Composition May Speed Resistance Evolution of Pests to Bt Crops

**DOI:** 10.1371/journal.pone.0169167

**Published:** 2017-01-03

**Authors:** Anthony R. Ives, Cate Paull, Andrew Hulthen, Sharon Downes, David A. Andow, Ralph Haygood, Myron P. Zalucki, Nancy A. Schellhorn

**Affiliations:** 1 Department of Zoology, University of Wisconsin-Madison, Madison, Wisconsin, United States of America; 2 CSIRO, Brisbane, Queensland, Australia; 3 CSIRO, Narrabri, New South Wales, Australia; 4 Department of Entomology, University of Minnesota, St. Paul, Minnesota, United States of America; 5 Ronin Institute, Montclair, New Jersey, United States of America; 6 School of Biological Sciences, The University of Queensland, St Lucia, Queensland, Australia; Institut Sophia Agrobiotech, FRANCE

## Abstract

Transgenic crops that express insecticide genes from *Bacillus thuringiensis* (Bt) are used worldwide against moth and beetle pests. Because these engineered plants can kill over 95% of susceptible larvae, they can rapidly select for resistance. Here, we use a model for a pyramid two-toxin Bt crop to explore the consequences of spatio-temporal variation in the area of Bt crop and non-Bt refuge habitat. We show that variability over time in the proportion of suitable non-Bt breeding habitat, *Q*, or in the total area of Bt and suitable non-Bt habitat, *K*, can increase the overall rate of resistance evolution by causing short-term surges of intense selection. These surges can be exacerbated when temporal variation in *Q* and/or *K* cause high larval densities in refuges that increase density-dependent mortality; this will give resistant larvae in Bt fields a relative advantage over susceptible larvae that largely depend on refuges. We address the effects of spatio-temporal variation in a management setting for two bollworm pests of cotton, *Helicoverpa armigera* and *H*. *punctigera*, and field data on landscape crop distributions from Australia. Even a small proportion of Bt fields available to egg-laying females when refuges are sparse may result in high exposure to Bt for just a single generation per year and cause a surge in selection. Therefore, rapid resistance evolution can occur when Bt crops are rare rather than common in the landscape. These results highlight the need to understand spatio-temporal fluctuations in the landscape composition of Bt crops and non-Bt habitats in order to design effective resistance management strategies.

## Introduction

Transgenic maize, potatoes, and cotton that carry the insecticidal toxins of *Bacillus thuringiensis* (Bt) are grown extensively throughout the world. Many of these Bt crops are highly effective against lepidopteran and beetle pests, and their adoption has considerably reduced the use of conventional chemical insecticides in some areas [1–5]. Nonetheless, they risk losing efficacy if targeted pests evolve resistance. The vast majority of theoretical work on evolution of resistance to Bt crops has explicitly or implicitly addressed spatial variation in Bt crops on the landscape: how much of the landscape is covered by Bt crops, and how the crops are arranged [6–9]. This work has generally assumed that spatial variation does not change through time, and little theoretical attention has been given to spatio-temporal variability in the risk of resistance evolution. Nonetheless, spatio-temporal variation in selection for Bt resistance could occur over the course of a year or among years, and could be driven by variation in the proportion of pest habitat that is planted to Bt crops or by variation in the total amount of pest breeding habitat, Bt-crops or otherwise. Here, we analyze how these types of spatio-temporal variation affect the risk of resistance evolution.

Current resistance management strategies for Bt crops are based on well-established assumptions about how resistance will likely arise. When there are high levels of expression of Bt proteins in transgenic crops and high toxicity, the most likely mode of resistance is through a single diallelic resistance gene for each toxin expressed by the crop, with resistance being recessive; in this "high dose" situation, resistance will probably be caused by loss of some function within the biochemical pathway attacked by the Bt toxin [10, 11]. The expectation that resistance to a single Bt toxin occurs through a single recessive allele is borne out by most documented cases of resistance to high-dose Bt crops [12]. Although the first-generation Bt crops expressed only a single Bt toxin, the second-generation Bt crops are "pyramids" containing two or more toxins which should, if both express high toxicity, make pyramid Bt crops more durable against resistance evolution [13–19]. When these toxins kill insects through different biochemical pathways, insect resistance requires the acquisition of resistance alleles at multiple genetic loci, and this can slow resistance evolution considerably [13, 16, 17, 19–22].

The cornerstone of resistance management is the high dose-refuge strategy in which monocultures of Bt crops are planted alongside refuge fields that contain non-Bt host plants [10, 23, 24]. This reduces the rate of resistance evolution mainly by reducing the proportion of the insect population that is killed by Bt, thereby reducing the strength of selection for Bt resistance [8, 25]. In theory, the high dose-refuge strategy can greatly reduce the rate of resistance evolution [7, 26–28], and its widespread use has been credited for the absence of more cases of insects evolving resistance [29]. Nonetheless, cases of resistance evolution to Bt crops are increasing [12], demanding continued re-evaluation of resistance management strategies.

**Fig 1:**
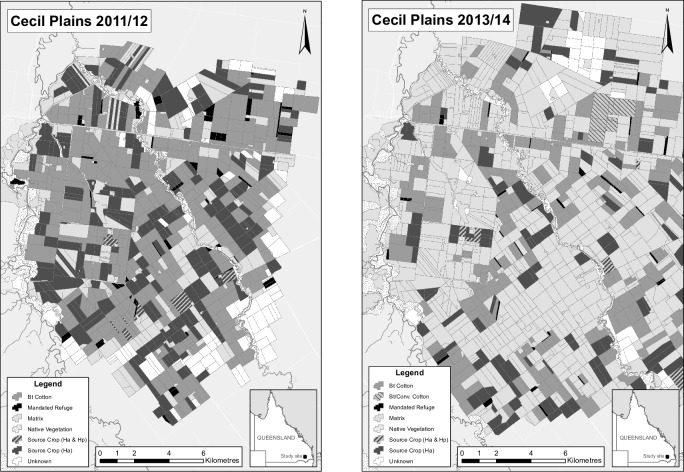
Landscape composition of the Cecil Plains, Queensland, Australia, study site in the 2011/12 and 2013/14 cropping seasons. Bt cotton is gray; mandated refuge is black; crops that provide breeding sites for both *H*. *armigera* and *H*. *punctigera* (source crops) are gray with dark-gray stripes while source crops for *H*. *armigera* alone are dark gray; non-breeding habitat (matrix) is light gray; native vegetation is stippled; and unknown habitat is white. In 2013/14 a few fields were planted with conventional cotton as mandated refuges within a more-extensive crop of Bt cotton (light gray with narrow stripes).

Spatio-temporal variation in the strength of selection for resistance to Bt could occur through year-to-year or within-season changes in the area of Bt crops and/or refuge habitat, either refuge habitat that is mandated for resistance management or unmandated refuge consisting of host plants in agricultural or native habitats. Here, we collectively call mandated and unmandated refuge simply refuge. To summarize spatio-temporal variation, we consider both the proportion of potential breeding habitat (Bt crops plus refuge) that is refuge, which we denote *Q*, and the total area of potential breeding habitat, which we denote *K*. For example, Australian cotton is attacked by two lepidopteran pests, the bollworms *Helicoverpa armigera* and *H*. *punctigera*. Bt cotton was rapidly adopted to control these pests and comprised over 90% of all cotton by 2006, although the area of cotton production has fluctuated greatly since 1998 [30]. For the agricultural landscapes of the cotton and grain-growing region of Cecil Plains, Darling Downs, Queensland, changes in climatic conditions (rainfall) and market prices among the years 2009–2014 caused the proportion of potential breeding habitat that was refuge for *H*. *armigera*, *Q*, to vary between 36% and 63%, and the proportion of potential breeding habitat on the landscape, *K*, to vary between 10% and 28%. For *H*. *punctigera*, *Q* varied between 18% and 38%, and *K* varied between 6% and 19% (Fig 1). (These values of *Q* and *K* were calculated by weighting different breeding habitats according to the oviposition preferences of *H*. *armigera* and *H*. *punctigera*; see Materials and Methods.) In addition to year-to-year variation in Bt crop and refuge area, there is seasonal variation: summer crops are planted in spring and harvested in fall, and winter crops are planted in autumn and harvested in late spring. Because the major winter crops are wheat and barley, which are poor hosts for *H*. *armigera* and not hosts for *H*. *punctigera*, this cropping seasonality leaves a 2–3 month period of low *K* in winter. Both *Helicoverpa* species have up to six generations per year in this region and can be active during the winter [31]; therefore, they experience large seasonal fluctuations in both Bt crops and refuges. Does this year-to-year and seasonal variation in Bt crop and refuge affect the rate of resistance evolution? If so, what risks does this pose to resistance management?

We investigated the consequences of spatio-temporal fluctuations in selection on resistance evolution using models for a two-toxin pyramid Bt crop. We first present a model tailored for the two bollworm pests of Australian cotton landscapes. This allows us to address general expectations about spatio-temporal variation in resistance and also specific management issues concerning these pests. In particular, we address the efficacy of "planting windows" that are designed to synchronize planting and harvesting of cotton, and thereby limit the selection on *Helicoverpa* to only three of six possible generations per year. In addition to the model tailored for *Helicoverpa* and the Australian landscapes, we also present a general model of resistance evolution for a generic pest that shows how the results from the *Helicoverpa* model may generalize to other pests and crop systems.

## Materials and Methods

### Insect biology and resistance

*Helicoverpa armigera* and *H*. *punctigera* are common pests of cotton in Australia, and while *H*. *punctigera* is a native [32], *H*. *armigera* now has a worldwide distribution [33]. Adults are capable of long-distance migration [34], but most *H*. *armigera* within agricultural landscapes disperse <10 km, whereas *H*. *punctigera* often fly >10 km [35]. Emerged females produce a sex pheromone that attracts potential male partners, and females may mate with multiple partners over the course of their lives [36, 37]. Although females may produce up to 1500 eggs in a lifetime [38, 39], most of these die in a density-independent manner, so we assume that females produce *F* = 50 surviving eggs; thus, the greatest possible increase in the population size from one generation to the next is 25 (after accounting for a 50:50 sex ratio). Finally, *H*. *armigera*, and to a lesser extent *H*. *punctigera*, may enter diapause in the pupal stage towards the end of the summer depending on temperature and light [40].

In addition to cotton, *H*. *armigera* has a broad host plant range which includes some native species but mostly exotics (e.g., the introduced weed Paterson’s curse, *Echium plantagineum*) and crops often planted near cotton: pigeon and chick peas, sorghum, maize, and to a much lesser extent cereals (wheat, barley) [41, 42]. Preference of *H*. *armigera* for several crops has been measured in bioassays [43–45]; when females are presented with chick peas, pigeon peas, maize, sorghum and cotton, the proportions of the females that oviposit are 0.98, 0.90, 0.70, 0.57, and 0.40 [45]. For cereals and native vegetation, we assume values of 0.10 and 0.01. The *H*. *punctigera* host plant range includes many natives (e.g., everlasting daisies, *Rhodanthe floribunda*) and introduced plant species; however, it is rarely found on monocotyledonous plants such as maize, sorghum, or cereals [32]. Therefore, we assume *H*. *punctigera* has the same preferences as *H*. *armigera* for the dicotyledonous crop plants but does not breed from monocots, and we assume that *H*. *punctigera* has a broader host range of native species (Table 1). Although we used fixed values for oviposition preference, we acknowledge that preferences change with crop stages, seasonal fluctuations, and climatic conditions.

**Table 1:** Baseline *Helicoverpa* preferences and seasonal availability for different habitat types.

	preference	preference	gen 1	gen 2	gen 3	gen 4	gen 5	gen 6
Habitat	*H*. *armigera*	*H*. *punctigera*	15 Oct	1 Dec	15 Jan	1Mar	15 May	15 Aug
Bt cotton	0.40	0.40	0	1	1	1	0	0
Conventional cotton	0.40	0.40	0	1	1	1	0	0
Pigeon pea	0.90	0.90	0	1	1	1	0	0
Chick pea	0.98	0.98	1	1	0	0	0	0
Maize	0.70	0	0	2/3	2/3	2/3	2/3	0
Sorghum	0.57	0	0	2/3	2/3	2/3	2/3	0
Cereal	0.10	0	1	0	0	0	0	1
*H*. *armigera* native	0.01	–	1	1	1	1	1	1
*H*. *punctigera* native	–	0.05	1	1	1	1	1	1

Values for preferences are based on oviposition lab bioassays of Jallow and Zalucki [44]. Values for seasonal availability are the proportion of the total annual area of habitat available to each of the six possible generations of *Helicoverpa*.

Densities of larvae in refuges will be substantially higher than in Bt fields, and this will likely lead to higher mortality rates due to density-dependent predation and parasitism, cannibalism, and, for unmandated crop refuges, insecticide or virus applications used to control outbreaks of *Helicoverpa* and other pests. For models including density-dependent larval survival, we assume that the relative strength of density dependence varies among habitats according to the preference that females show for oviposition. Thus, since the preference of *H*. *armigera* females for chick peas is 2.45 (= 0.98/0.40) times greater than for cotton, we assume that the survival of larvae in chick peas is the same as the survival in cotton when the density of eggs oviposited in chick peas is 2.45 times that in cotton. This assumption means that the potential production of pupae from different habitats scales with the oviposition preference that females show for these crops; a field of chick peas can produce the same number of adult moths as a cotton field that is 2.45 times larger. This scaling only affects the relative strength of density dependence among habitats. In the model, the overall strength of density-dependent mortality is determined by the population density: at the population level, the strength of density dependence is whatever is required to give a long-term per capita population growth rate of zero.

Since 2004 roughly 80% of the cotton planted in Australia has been Bollgard II which protects against *Helicoverpa*. Bollgard II is a two-toxin pyramid consisting of Cry1Ac and Cry2Ab. While the toxicity of these two toxins varies through the growing season (cotton development stage), Cry1Ac is generally the less toxic, and we use relative survivals of susceptible *H*. *armigera* and *H*. *punctigera* genotypes of *s*_1SS_ = 0.15 and *s*_2SS_ = 0.05 for Cry1Ac and Cry2Ab, respectively. Because both toxins are expressed in moderate to high doses, resistance is expected to take the form of single diallelic genes that are largely recessive; we assume a dominance of *h* = 0.05, leading to relative survivals of resistance-susceptible (RS) genotypes on single toxin plants equal to *h s*_1RR_ + (1–*h*) *s*_1SS_ = 0.1925 and *h s*_2RR_ + (1–*h*) *s*_2SS_ = 0.0975 for Cry1Ac and Cry2Ab, respectively; we assume that resistance of RR individuals is complete, so *s*_1RR_ = *s*_2RR_ = 1. Finally, for the two-toxin pyramid plants, we assume that survivals are independent (multiplicative), as is expected if toxins have independent modes of action [46]. For example, the survival of an S_1_S_1_S_2_S_2_ individual on Bt plants is *s*_SSSS_ = *s*_1SS_ × *s*_2SS_ = 0.0075, and the survival of an R_1_S_1_R_2_S_2_ individual is *s*_1RS_ × *s*_2RS_ = 0.0188. In the cotton-growing region of Australia the current estimated frequencies of alleles conferring resistance against Cry1Ac and Cry2Ab are 0.01 and 0.003, respectively, for *H*. *punctigera*, and 0.02 and <0.001 respectively for *H*. *armigera* (based on F1 screens: [4, 47] and Downes, unpublished data).

### Landscapes

Cotton-growing regions of Australia are characterized by a complex mixture of different crops and land uses (Fig 1). A key component of the Bollgard II resistance management plan in Australia is that unsprayed conventional (non-Bt) cotton or pigeon pea must be planted as mandated refuges, at no less than 10% and 5%, respectively, of the area of Bt cotton. In addition to mandated refuges, a considerable area may be planted in non-Bt crops that serve as unmandated refuges, especially for *H*. *armigera* that has the broader host range of crop species (Table 1). A second part of the resistance management plan consists of "planting windows" in which Bt cotton and mandated refuges are planted within a specified 6-week period during the spring. Planting windows are required in latitudinal regions where the relatively aseasonal climate would allow, in the absence of planting windows, crop production year round; elsewhere, only an end date to planting is imposed since its start is restricted by climate [48]. Planting windows are primarily designed to limit the exposure of pests to Bt cotton by leaving a 2 to 3-month period in which Bt fields remain fallow; in principle, this means that there is no selection for resistance during the last two of the possible six annual insect generations per year at our study sites.

We used cotton-growing landscapes from three study sites (Cecil Plains, Nandi, and Pampas) in the Darling Downs situated 200 km west of Brisbane, Queensland, Australia. The three landscapes are each approximately 20 km in diameter and separated by approximately 35 km. Land-use information was characterized for five summer cropping seasons, December-March, from 2009/10 to 2013/14. Crop varieties and planting dates were collected by contacting individual growers from each region every season, and confirmed by visiting fields and ground-checking the details. Field boundaries of crop and non-crop habitat were digitized using the satellite image service base map in ArcGIS 10.2 [49]. Land parcels in the landscape maps were partitioned into 27 different use categories including roads, buildings, water storage and different crops and host plants. The areas of these land parcels were then used to calculate proportions of characteristic land use, host plants, and breeding habitat for *H*. *armigera* and *H*. *punctigera* for each landscape for each season.

### Model

The "*Helicoverpa* model" is designed around *H*. *armigera* and *H*. *punctigera* in the Darling Downs cotton-growing region. An important biological difference between species is that *H*. *armigera* uses a broader range of crops while *H*. *punctigera* can use native vegetation to a greater extent (Table 1); therefore, they have contrasting patterns of fluctuations in *Q* and *K*. In addition to the *Helicoverpa* model, we also analyzed a simplified "general model" that more clearly exposes the role of spatio-temporal variation on the rate of resistance evolution as it might affect other pests.

The *Helicoverpa* model uses the annual distribution of breeding habitat for each of six generations given in Table 1. So that we can focus on *in situ* resistance evolution of *H*. *punctigera*, we ignored the possibility of long-range migratory behavior into and out of the cotton-growing region [41, 50]. The possible consequences of immigration depend on the details of both the frequency of resistance alleles in the immigrant population and the timing of immigration which affects whether mating occurs [51]. Although immigration could change the rate of resistance evolution (e.g., through diluting resistance alleles), it will not change the impact of spatio-temporal variability and hence the qualitative results. Parts of generations 3–5 can delay development or enter diapause and emerge at the start of the following year; specifically, we assume that the proportions of the *H*. *armigera* population in diapause are 0.74, 0.95, and 0.95 in generations 3, 4, and 5; for *H*. *punctigera*, these are 0, 0.30, and 0.70.

We ran the *Helicoverpa* model using landscapes made up of Bt cotton, mandated refuges, and unmandated refuges in the same proportions as calculated for Cecil Plains, Nandi, and Pampas regions. To weight habitats by both oviposition preference and density dependence, we multiplied the area of each habitat by the oviposition preference value; for example, chick pea is weighted by 0.98, and cotton is weighted by 0.40. These weighted values were used to calculate both the proportion *Q* of the adult female population that oviposits in refuge habitats and the total *K* of the landscape that is potentially breeding habitat. Thus, a landscape made up of 5% chick pea and 80% Bt cotton would have *Q* = (0.05*0.98)/(0.05*0.98 + 0.80*0.40) = 0.13 and *K* = 0.05*0.98 + 0.80*0.40 = 0.37.

The models keep track of both allele frequencies and insect densities, and operate in discrete time with a single time step equal to a generation [8]. The order of life-history events within a generation is male movement among fields, mating, female movement and oviposition, selective mortality of larvae by Bt toxins in Bt fields, and density-independent and density-dependent mortality from sources other than Bt toxins. Mating within fields is assumed to be random, and movement is assumed to be “global”, independent of the spatial arrangement of fields.

We investigated density-dependent survival using three separate models: models L, A, and I. For model L (“larval density”) density-dependent larval survival is given by (1 + *a*_*t*_*x*)^–1^ where *x* is the density of larvae within a field, and *a*_*t*_ scales the relative strength of density dependence which can vary from one insect generation *t* to the next. This equation applies to both Bt and non-Bt habitats, although because the larval density in Bt cotton is reduced by mortality of susceptible larvae, the effect of density dependence is correspondingly less. To scale the total area of habitat, we use the population density that a completely resistant insect population would attain if *K* were constant; if the total availability of breeding habitat is scaled according to the size of the insect population it can support, this gives *a*_*t*_ = (*F*/2–1)/*K*_*t*_. If *X*_*B*_ and *X*_*R*_ represent the relative numbers of larvae in Bt and refuge habitats, then density dependence varies with the densities of larvae in Bt crops and refuges given by *X*_*B*_/(*K*_*t*_(1 –*Q*_*t*_)) and *X*_*R*_/(*K*_*t*_*Q*_*t*_), respectively. Thus, the density-dependent survivals of larvae in Bt crops and refuge are (1 + α*X*_*B*_/(*K*_*t*_(1 –*Q*_*t*_)))^–1^ and (1 + α*X*_*R*_/(*K*_*t*_*Q*_*t*_))^–1^, where α = (*F*/2–1).

For model A (“adult oviposition limitation”) we assumed the same effect of density dependence on larvae as in model L. In addition, female fecundity depends on the amount of available habitat. In model L all females lay *F* eggs, even if the total breeding habitat *K* decreases between generations; this would require all females to find whatever habitat remains. In model A the effective fecundity of females is proportional to the available breeding habitat, *F*_*t*_ = *FK*_*t*_/*K*_*max*_, where *K*_*max*_ is the maximum available breeding habitat. This reduction in fecundity is determined by the overall availability of breeding habitat including Bt crops. Therefore, it gives a density-dependent reduction in the population growth rate for the entire population. Because *Helicoverpa* and other pests of Bt crops have specialized mechanisms for finding breeding habitat, model A is unlikely to be realistic. On the other hand, model L is also likely to be unrealistic when there are large decreases in the amount of breeding habitat each generation. Therefore, models L and A represent extremes of a continuum, with *Helicoverpa* likely to fall towards the side of model L.

For model I (“independent of density”), there is no density dependence; the model only keeps track of allele frequencies, not densities. The genetic component of the model is identical to models L and A. Matlab code [52] for the models is available in S1 File.

#### Quantifying selection

Investigating the effects of variation in *Q* and *K* on the rate of resistance evolution requires a generation-to-generation measure of the strength of selection. The strength of selection does not depend on specific allele frequencies in a given generation or the level of dominance *h* expressed by heterozygotes. Therefore, it gives a way to calculate the effects of spatio-temporal variation on selection in the models that does not depend on the genetic details of the population. We derive this measure here, although this section can be skipped if the technical details are not needed.

The strength of selection is determined by Ф_*t*_, the survival of completely resistant genotypes to Bt toxins relative to the survival of susceptible genotypes in generation *t*. For model L, the number of homozygous susceptible larvae that survive Bt toxins within Bt crops is *s*_SSSS_ *X*^*sB*^_*t*_, where *X*^*sB*^_*t*_ is the number of homozygous susceptible larvae in Bt crops before mortality caused by Bt toxins, and *s*_SSSS_ is the survival of susceptible homozygotes to Bt toxins. The number of homozygous susceptible larvae that survive Bt toxins because they occur in refuge habitat is *X*^*sR*^_*t*_ (1 + *a*_*t*_ *X*^*sR*^_*t*_ /*Q*_*t*_)^–1^) where *X*^*sR*^_*t*_ is the number of homozygous susceptible larvae within refuges. The strength of selection Ф_*t*_ is the number of homozygous susceptible larvae in the total population before mortality caused by Bt divided by the number after mortality from Bt:

$$\Phi_{t}=\frac{X_{t}^{sB}+X_{t}^{sR}{\left(1+a_{t}X_{t}^{sR}/Q_{t}\right)}^{-1}}{s_{ssss}X_{t}^{sB}+X_{t}^{sR}{\left(1+a_{t}X_{t}^{sR}/Q_{t}\right)}^{-1}}$$ (1)

To confirm that the strength of selection correctly predicts the rate of resistance evolution, it can be used to calculate the characteristic time to control failure, *T*_*c*_:

$$T_{c}=\frac{1}{E\left[log\left(1-h_{1}h_{2}+h_{1}h_{2}\Phi_{t}\right)\right]}$$ (2)

For the case in which the initial frequency of an allele is *p*_0_ and control failure occurs at *p*_*f*_, the total time to control failure is *T*_*c*_ log(*p*_*f*_/*p*_0_). Furthermore, provided the expression of resistance by heterozygotes is small *E*[log(1—*h*_1_*h*_2_ + *h*_1_*h*_2_Ф_*t*_)] ≅ *h*_1_*h*_2_*E*[Ф_*t*_]. Therefore, the rate of resistance evolution is approximately proportional to *E*[Ф_*t*_]. This shows that Ф_*t*_ can be used to identify generations *t* that are responsible for strongest selection for resistance.

## Results

We first investigate the cases of *H*. *armigera* and *H*. *punctigera* on the Darling Downs using our data on observed variation in habitat for 2009/10-2013/14. We then address the issues of variation in *Q* and *K* more broadly using the general model. The results for *H*. *armigera* and *H*. *punctigera* are self-standing, and the general model serves to address the same issues conceptually for the case of a generic system.

### *Helicoverpa* model

For the *Helicoverpa* model, we measured the rate of resistance evolution as the time to resistance failure (>50% individuals are resistant) for Cry2Ab, which is more toxic than Cry1Ac. Resistance to both Cry2Ab and Cry1Ac occur at similar times, because once resistance arises to one toxin, Bollgard II is no longer a two-toxin pyramid. Because resistance can take more than six years, we repeated our 6-year landscape data so that year 7 has the same proportions of crops and other vegetation on the landscape as year 1, etc. We parameterized the model for both *H*. *armigera* and *H*. *punctigera* using three landscapes in the Darling Downs. Here we present the results for the Cecil Plains landscape, with parallel results for Nandi and Pampas in the Supplementary Information (S1 and S2 Figs).

We considered the case in which there is a strict planting window for Bt cotton and mandated refuges (conventional cotton and pigeon pea), so that these crops are only available to *H*. *armigera* and *H*. *punctigera* for generations 2–4 (Table 1). A central concern for resistance management is the effective maintenance of the planting window that confines selection to only generations 2–4. Early planting or late emergence of generation 1 could expose insects in that generation to selection, while late harvesting or early emergence of generation 5 could expose that generation to selection. Therefore, we compared the case of strict planting windows to the case in which 10% of Bt and refuge (conventional) cotton was available in generation 1, or 10% of Bt and refuge cotton was available in generation 5.

**Fig 2:**
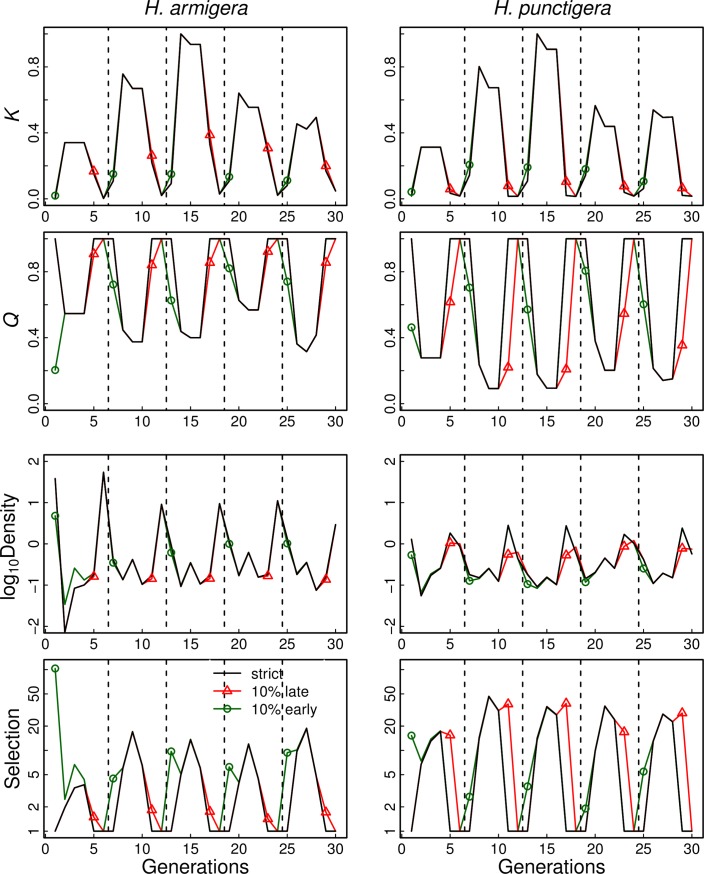
For model L, selection for resistance in *H*. *armigera* and *H*. *punctigera* under three scenarios about crop planting: (i) cotton (Bt and conventional) is only available in generations 2–4 (black lines); (ii) like (i) but 10% of cotton is also available for generation 5 (red lines and triangles); and (iii) like (i) but 10% of cotton is also available for generation 1 (green lines and circles). The relative contributions of different crops and native vegetation for each of six years are based on field data from Cecil Plains. The top panels give *K*, the total available breeding habitat (Bt cotton, mandated refuges, and unmandated refuges) and the next-lower panels give *Q*, the proportion of breeding habitat that is refuge. Years are demarcated by vertical dashed lines. The 6-year patterns in *Q* and *K* are repeated in the simulations, so the values in first generation (first point on the left) arise from the values in the thirtieth generation (last point on the right). The next-lower panels are the log_10_ densities of eggs in refuges, measured before density-dependent mortality of larvae. The bottom panels give the strength of selection for resistance, Ф_*t*_ (Eq 1). For all simulations, *F* = 50, and habitat preferences are given in Table 1.

Considering first the case of model L and strict planting windows, there are strong seasonal patterns in the total available breeding habitat, *K*, and the proportion of breeding habitat that is mandated and unmandated refuge, *Q* (Fig 2, black lines). These generate a strong seasonal pattern for the density of eggs laid in refuges. High egg and larval densities increase density-dependent mortality of larvae, which in turn decreases the fitness of susceptible larvae in refuges relative to the fitness of resistant larvae in Bt fields. This leads to strong selection. For *H*. *armigera*, the highest density of eggs in refuges occurs in generation 6, which is caused by the drop in *K* and hence the limited habitat for female oviposition. There is also a secondary peak in egg density in generation 3; the density is greater than in generation 2 due to the population increase with high *K*, while in generation 4 the egg density decreases due to larvae entering diapause at the end of generation 3. For *H*. *punctigera*, peak densities occur in generation 5, because it does not breed on sorghum and maize that are available to *H*. *armigera*. For both species, selection for resistance spikes in generation 3 due to little refuge (low *Q*) and higher density of larvae in refuges. These spikes in selection are particularly high for *H*. *punctigera*, because it has less refuge (lower *Q*) than *H*. *armigera*.

If 10% of cotton (both Bt and convention) remains available in generation 5, there is strong selection for resistance in *H*. *punctigera* (Fig 2, red lines) because cotton represents 40–80% of its breeding habitat (*Q* = 0.6–0.2) and generation 5 experiences high larval densities within refuges due to relatively low total breeding habitat, *K*. In contrast, *H*. *armigera* is little affected by late-season unharvested cotton, because >95% of the population in generation 5 is in diapause, leading to little exposure to Bt and low densities within refuges.

If 10% of cotton becomes available in generation 1, this generates strong selection for resistance in *H*. *armigera* (Fig 2, green lines). For *H*. *armigera* there is little unmandated refuge in generation 1, so even if only 10% of the cotton crop is available, *Q* < 0.8, and in the first year of data (2009) *Q <* 0.2. Furthermore, the very low value of *K* in the first generation of 2009 leads to high densities of eggs in the little available breeding habitat, causing a high spike in selection. The low *Q* in 2009 is due to the absence of chick pea planting. Because this single spike in the first generation of 2009 appears to dominate selection over the 6-year period, we also modeled the case in which the values of *Q* and *K* for 2009 were replaced by the values from 2013 (S3 Fig); this replacement caused little change in the overall rate of resistance evolution (S4 Fig). Finally, in contrast to *H*. *armigera*, for *H*. *punctigera* there is little effect of early cotton on selection due to their low densities in refuges.

**Fig 3:**
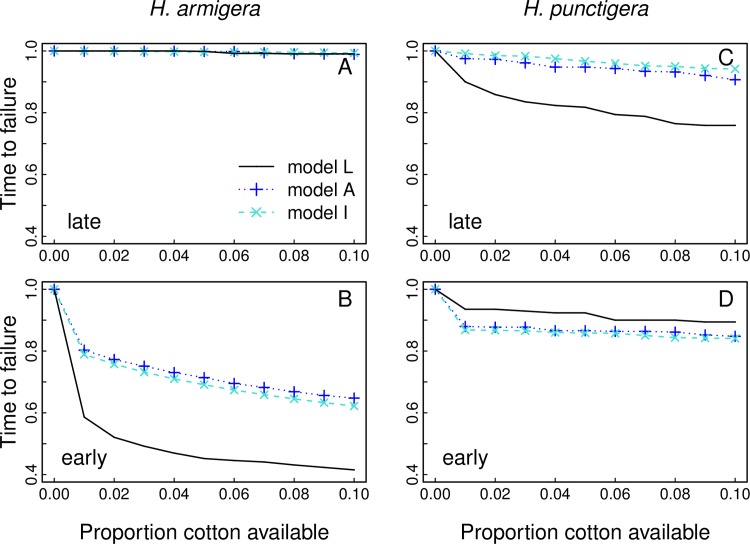
**Relative time to control failure for *H*. *armigera* (A,B) and *H*. *punctigera* (C,D) versus the proportion of cotton crop (Bt and conventional) that is either (A,C) available late to generation 5 or (B,D) available early to generation 1.** Results are for model L (black lines), model A (dashed blue lines and +'s) and model I (turquoise dashed lines with x's). Time to control failure is given relative to the case of strict cotton cropping that is available to only generations 2–4, with time to control failure given when the allele frequency of resistance to Cry2Ab reaches 0.5. Habitat proportions are given by the landscape at Cecil Plains for 2009–2014, and the habitat preferences for each species are the same as Fig 2.

The role of density-dependent larval mortality in resistance evolution can be shown by comparing the results from model L with those from models A and I (Fig 3). Models A and I also show sizable effects of cotton harvested late or planted early. Late or early cotton not only affects densities of larvae in refuges (which has little effect in model A and no effect in model I), but also influences *Q*. Indeed, for *H*. *armigera* 10% early cotton can halve the time to resistance failure in models A and I, even though this occurs in only 1 of 6 generations per year and only involves 10% of the Bt cotton. Another feature of all three models is that much of the increase in resistance evolution occurs at very low levels of early or late cotton: 1% early or late cotton leads to loss of durability (time to resistance failure) that is roughly half the loss that occurs with 10% early or late cotton. Finally, although larval density dependence exacerbates the effects of early cotton for *H*. *armigera* and late cotton for *H*. *punctigera* (compare model L with models A and I), the opposite is true for the effect of early cotton on *H*. *punctigera* (Fig 3D) in which models A and I show greater reductions in the time to resistance failure than model L. The explanation for this is that, in model L, early cotton causes a decrease in *H*. *punctigera* density in refuges in generation 1, because early cotton provides more total breeding habitat (Fig 2); this mitigates the increase in selection caused by reducing *Q*. In contrast, in models A and I there is no release from selection caused by the increase in density that occurs in model L (S2 Fig). This specific result in which model L shows less sensitivity to early cotton than models A and I illustrates the possible importance of knowing the scale at which density dependence occurs, at the field scale (model L), at the landscape scale (model A), or not at all (model I).

The results in Fig 3 are given in terms of relative changes in times to resistance failure; the relative times to resistance are insensitive to initial allele frequencies provided they are moderately low (< 0.01). The models also differ in absolute times to resistance failure, with density dependence reducing times to resistance [8]. Specifically, for *H*. *armigera* with initial allele frequencies of 0.01 for Cry1Ac and 0.003 for Cry2Ab, resistance occurs in 188, 296, and 998 generations for models L, A, and I when the proportion of early or late cotton available is 0. For *H*. *punctigera* with initial allele frequencies of 0.02 for Cry1Ac and 0.001 for Cry2Ab, resistance occurs in 81, 152, and 339 generations for models L, A, and I.

### General model

**Fig 4:**
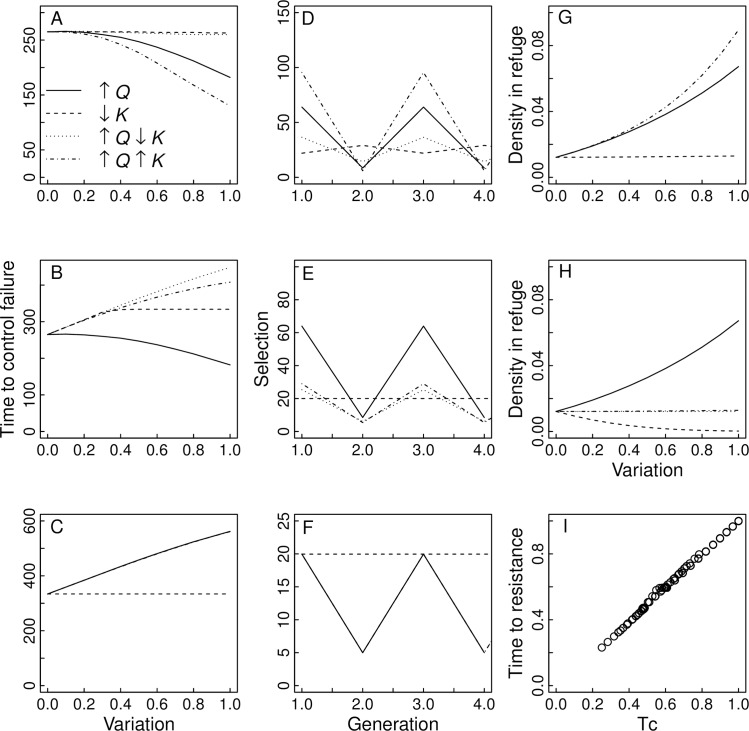
For models L, A and I, the effects of variation in *Q* and/or *K* for four scenarios: (i) only *Q* varies, with *Q*_1_ = 0.05 and *Q*_2_ ranging from 0.05 to 0.2 (indicated on the horizontal axis labeled "Variation" which is scaled from 0 to 1 as *Q*_2_ goes from 0.05 to 0.2), while *K*_1_ = *K*_2_ = 1 (solid line); (ii) only *K* varies, with *K*_1_ = 1 and *K*_2_ in the ranging from 1 to 0.25 (indicated by values of "Variation" ranging from 0 to 1), while *Q*_1_ = *Q*_2_ = 0.05 (dashed line); (iii) *Q* and *K* vary asynchronously, with *Q*_1_ = 0.05 and *Q*_2_ ranging from 0.05 to 0.2, and *K*_1_ = 1 and *K*_2_ ranging from 1 to 0.25 (↑*Q*↓*K*, dotted line); and (iv) *Q* and *K* vary synchronously, as in (iii) but with high values of *Q*_2_ coinciding with high values of *K*_2_ (↑*Q*↑*K*, dot-dashed line). (A-C) give the times to control failure (when resistance allele frequency reaches 0.5) for models L, A, and I, respectively. (D-F) give the strength of selection Ф_*t*_ (Eq 1) for four consecutive generations for models L, A and I, respectively, and for scenarios (i)—(iv). (G-H) give the densities of eggs (numbers/[*Q*_*t*_*K*_*t*_]) in refuges before density-dependent mortality and averaged over both generations for models L and A, respectively. (I) The predicted relative time to control failure from the approximation giving *T*_*c*_ (Eq 2) and the observed time to control failure for all of the simulated runs for all three models shown in (A-C). Other parameter values are: *F* = 50, *s*_1_ = 0.01, *s*_2_ = 0.01, and *h*_1_ = *h*_2_ = 0.05.

To illustrate the effect of variation in *Q* and *K* for the general case of an unspecified pest, we built general models designed to clarify the roles of spatio-temporal variation on resistance evolution. The general models are similar to the *Helicoverpa* models but have *s*_1SS_ = *s*_2SS_ = 0.01 and *h*_1_ = *h*_2_ = 0.05. We investigated the cases for models L, A, and I in which *Q* varies by itself, *K* varies by itself, *Q* and *K* vary synchronously (high *Q* occurs with high *K*), and *Q* and *K* vary asynchronously. Variation in *Q* is generated by fixing *Q*_1_ = 0.05 and increasing *Q*_2_ up to 0.2, while variation in *K* is generated by fixing *K*_1_ = 1 and decreasing *K*_2_ down to 0.25. Fig 4 shows the time to resistance failure, the strength of selection Ф, and the density of eggs in the refuge before density dependence occurs. The fact that Ф gives a good measure of the strength of selection can be seen by plotting the simulated rate of resistance evolution against the rate predicted from Ф (Eq 2) for all of the models and cases depicted in Fig 4A–4C (Fig 4I).

Considering first the case of variation in *Q* (Fig 4, solid lines), for model I increasing variation increases the time to resistance (Fig 4C). This occurs because increasing variation in *Q* by increasing *Q*_2_ increases the mean of *Q* from 0.05 to 0.1 when *Q*_2_ = 0.2; variation in *Q* if the mean is kept the same has no effect on resistance evolution in model I. In contrast, in model L increasing variation in *Q* decreases the time to resistance. This is caused by a spike in the strength of selection over 60 (Fig 4D), compared to 20 in model I (Fig 4F). The greater selection in model L occurs because the large number of females produced when *Q*_2_ is high leads to high density of eggs in the following generation when *Q*_1_ is low; the resulting increase in density-dependent mortality of larvae in refuges decreases the survival of susceptible larvae and increases the relative fitness of resistant larvae. Model A shows the same results of varying *Q* as model L, because in model A females cannot distinguish between refuge and Bt fields, and therefore they lay the same number of eggs as in model L.

Variation in *K* (Fig 4, dashed lines) has no effect in model I, since this model does not include density. In model L it also has little effect on the overall rate of resistance evolution (Fig 4A) and the density in refuges (Fig 4G), even though it leads to fluctuations in selection (Fig 4D). Selection increases in the generation when *K* is low, because this decreases the density in refuges and hence density-dependent mortality. Nonetheless, in the following generation when *K* is high, the density in the refuge is low so selection decreases, balancing out the higher selection in the preceding generation. In model A, variation in *K* increases the time to resistance (Fig 4B). This occurs because when *K* decreases, the number of eggs produced per female decreases, leading to lower average densities in the refuge. When densities drop to very low levels at high variation in *K*, there is little density dependence, and model A gives the same times to resistance (Fig 4B) and selection (Fig 4E) as model I that contains no density dependence.

When both *Q* and *K* vary, their net effect depends on whether they vary synchronously or asynchronously, and on the model. For model I, because variation in *K* has no effect, the results when both *Q* and *K* vary are identical to the results when only *Q* varies (Fig 4C and 4F). For model L, when *Q* and *K* vary asynchronously (Fig 4, dotted lines), their effects on selection (Fig 4D) largely cancel each other out, because the reduction in the proportion of refuge when *Q* is low is compensated for by the increase in total area of breeding habitat when *K* is high. Thus, there is little change in the density in refuges (Fig 4G; the dotted line and the solid line for only *Q* varying are almost identical). In contrast, when *Q* and *K* vary synchronously (Fig 4, dot-dashed lines) their effects are compounded, leading to both rapid resistance and high average densities in the refuge. Although for model L synchronous fluctuations in *K* compound the effect of variation in *Q*, in model A the effects of synchronous fluctuations in *Q* and *K* are intermediate between the cases of fluctuations in only *Q* and only *K*. This illustrates the possible complexities arising from fluctuations in both *Q* and *K*, with their combined effects depending on how pests respond behaviorally to changes in breeding habitat *K*.

## Discussion

Our results lead to two broad conclusions about resistance evolution in a spatio-temporally fluctuating environment. First, temporal variation in the proportion of refuge (non-Bt) habitat *Q* and total breeding habitat *K* can lead to brief but intense bursts of selection, and the overall rate of resistance evolution can be determined largely by the surges in resistance that these short bursts of selection generate. Second, the intensity of these bursts of selection can be exacerbated by density-dependent mortality if (i) the bursts of selection coincide with bottlenecks in the availability of breeding habitat and (ii) as a result insects in refuges experience an episode of greater-than-normal density-dependent mortality. Thus, when there is potential density-dependent mortality in refuges, there can be an interaction between changes in the proportion of breeding habitat that is refuge and changes in the density of the pest insect that combine to increase surges in resistance evolution and diminish the durability of a Bt crop.

Temporal variation in *Q* and/or *K* affect the rate of resistance evolution by causing large numbers of insects generated in one generation to be crowded into small areas of remaining non-Bt habitat in the next generation. This crowding will have little effect on rare resistant insects in Bt fields, because most susceptible larvae will be killed by Bt toxins, thereby reducing densities and density-dependent mortality. However, larvae within refuges, most of which are susceptible, will suffer higher density-dependent mortality, resulting in higher survival of resistant larvae in Bt crops relative to the rest of the populations, and consequently more rapid resistance evolution.

We investigated the role of larval density dependence by comparing three models. In model L (larval density) females deposit all of their eggs, and therefore larval density dependence is the only form of density dependence in the population. In model L, variation in *Q* or *K* alone can speed resistance evolution, and when they vary in synchrony, the time to resistance evolution can speed up greatly. In model A (adult oviposition limitation) females deposit their eggs in proportion to the availability of breeding habitat; thus, if *K* drops from 1 to 0.25, fecundity drops by 75%. In contrast to larval mortality in refuges, this density dependence acts uniformly across the entire population, and because it is the same for resistant and susceptible genotypes, it has no direct effect on resistance evolution. Because female pests (including *Helicoverpa*) targeted by Bt crops are able to locate suitable breeding habitat, model A represents an unlikely extreme, although models L and A likely bracket reality. In model A, variation in *Q* has the same effect as in model L, because it does not change the proportion of breeding habitat. However, the effect of variation in *K* in model A slows resistance evolution because on average it reduces the fecundity of females, which reduces the difference in densities between Bt crops and non-Bt refuges. In model I that has no larval density dependence, variation in *Q* and *K* have no effect.

In practice, understanding the consequences of temporal variation in Bt crops, mandated refuges, and unmandated refuges is more complicated. *Helicoverpa armigera* and *H*. *punctigera* on Bt cotton in Australia experience year-to-year and seasonal variation in *Q* and *K*. To investigate the consequences of this variation, we focused on resistance management when there is the possibility of cotton being available either before or after planting windows. Even though these times early or late in the growing cycle when *Helicoverpa* are be exposed to Bt might be short, they could still drive surges in selection for resistance. This is because, in terms of larvae killed by Bt, the strength of selection for resistance depends not on total numbers but instead on proportions. If the overall population is small, a small number of larvae killed by Bt could still be a large proportion of the population on the landscape.

The complications of spatio-temporal variation in cropping schedules are highlighted by comparing results for *H*. *punctigera* and *H*. *armigera* (Figs 2 and 3) that differ in their use of crops and natural vegetation. Late cotton has the potential to greatly speed resistance evolution for *H*. *punctigera*, because with little unmandated refuge in generation 5, *Q* for *H*. *punctigera* is low. In model L, this is exacerbated by high *K* in generation 4 which leads to high densities of larval *H*. *punctigera* in refuges in generation 5 and consequent increased selection. In contrast, early cotton has a greater impact on *H*. *armigera* than *H*. *punctigera*, because little unmandated refuge for *H*. *armigera* in generation 1 leads to a larger proportional decrease *Q* in generation 1 compared to *H*. *punctigera*. In model L, this is exacerbated by relatively high densities of *H*. *armigera* larvae in refuges compared to *H*. *punctigera* in generation 1. Thus, the combination of differences among cropping phenologies and differences in suitable breeding habitat for the two species has a large impact on the consequences of imperfect planting windows.

What is the likely magnitude of density-dependent larval mortality in real systems? In models L and A, the effect of variation in *Q* and *K* on resistance evolution depends only on density dependence in the mortality of larvae, because selection for Bt resistance acts on this life stage. The key issue is the survival from egg to emergence of a resistant larva in a Bt field relative to the survival of a susceptible larva in the refuge. Density-dependent mortality of eggs will not affect resistance evolution if egg densities are the same in Bt crops and refuges, and density-dependent adult survival and/or fecundity will not affect resistance evolution because it will, like eggs, be independent of genotypes. Several sources of density-dependent larval mortality likely affect *Helicoverpa* within refuges. Many parasitoids and predators show density-dependent responses to larval prey. For example, *Microplitis* parasitoids and their associated viruses can cause >50% larval mortality of *H*. *armigera* and *H*. *punctigera* in Australian cotton [53, 54] and show increasing parasitism with host density at the scale of fields [55]. Similarly, viral and fungal pathogens often show density-dependent spread through fields [56]. *Helicoverpa*, especially *H*. *armigera*, are cannibalistic at high density which can cause significant increases in mortality [57, 58]. Finally, growers may cause density-dependent larval mortality in unmandated refuges if they take control measures against *Helicoverpa* when densities are high, or if they take control measures against other pests that show population increases synchronously with *Helicoverpa* and the latter are also killed.

The magnitude of the potential surges in selection seen in our *Helicoverpa* model if there is available Bt cotton in insect generations 1 or 5, will likely depend on factors that are not realistically incorporated into the model. For instance, the model assumes that insect generations are non-overlapping, and does not account for changes in female oviposition during harvesting and movement across the landscape which can affect whether eggs are deposited into crops with sufficient time to reach the pupal stage. Instead, we tried to bracket the extremes of the abilities of females to find breeding habitat using models L and A. Therefore, while the models show that surges in selection are possible, it is hard to anticipate the magnitude of these surges in reality.

Our results show that variation in the proportion of refuges *Q* or total habitat *K* can increase the rate of resistance evolution for other pests and Bt crop systems. For some Bt crops, variation in *Q* and *K* occur due to market forces and annual climatic fluctuations [59]. This variation can speed resistance evolution and can lead to counter-intuitive recommendations. For example, it might make apparent sense for growers to increase the amount of refuge in years when pest pressure is low, since this would relax selection for resistance. Our results, however, show that this strategy might speed resistance evolution if later reductions in refuge area cause a surge in resistance evolution. Thus, growers trying to do the right thing might end up speeding resistance evolution.

A particular concern highlighted by our results is that selection for resistance may be strongest when the area planted in Bt crops is small rather than large; the surges in selection we found in the models occurred when most of the Bt crop was not in the ground. Thus, the greatest risk of resistance evolution might occur when Bt crops are rare rather than common. Resistance management must be performed to avoid possible episodic surges in selection, although this may be a challenge in complex and variable agricultural systems. Nonetheless, as resistance failures would require reliance on less effective and more environmentally harmful pest control tactics, the costs of not meeting this challenge would be considerable.

## Supporting Information

S1 Fig For the Cecil Plains, Pampas, and Nandi landscapes, selection for resistance in *H*. *armigera* and *H*. *punctigera* under three scenarios about crop planting: (i) cotton (Bt and conventional) is only available in generations 2–4 (black lines); (ii) like (i), but 10% of cotton is also available for generation 5 (red lines and triangles); and (iii) like (i), but 10% of cotton is also available for generation 1 (green lines and circles). See Fig 2 in the main text. The relative contributions of different crops and native vegetation for each of six years are based on field data from Cecil Plains, Pampas, and Nandi study areas. The top panels give *K*, the total available breeding habitat (Bt cotton, mandated refuges, and unmandated refuges) and the next-lower panels give *Q*, the proportion of breeding habitat that is refuge. Years are demarcated by vertical dashed lines. The 6-year patterns in *Q* and *K* are repeated in the simulations, so the values in first generation (first point on the left) arise from the values in the thirtieth generation (last point on the right). The next-lower panels are the log_10_ densities of larvae in refuges, measured before density-dependent mortality. The bottom panels give the strength of selection for resistance, Ф_*t*_ (Eq 1). For all simulations, *F* = 50 and survival of diapausing pupae is 0.2. Habitat preferences are given in Table 1.
(TIFF)

S2 Fig **For the Cecil Plains, Pampas, and Nandi landscapes, the relative time to control failure for *H*. *armigera* (A,B) and *H*. *punctigera* (C,D) versus the proportion of cotton crop (Bt and conventional) that is either (A,C) available late to generation 5 or (B,D) available early to generation 1.** See Fig 3 in the main text. Results are for model L (black lines), model A (dashed blue lines and +'s) and model I (turquoise dashed lines with x's). Time to control failure is given relative to the case of strict cotton cropping that is available to only generations 2–4, with time to control failure given when the allele frequency of resistance to Cry2Ab reaches 0.5; resistance to Cry2Ab occurs before resistance to Cry1Ac. Habitat proportions are given by each landscape 2009/10-2013/14, and the habitat preferences for each species are the same as S1 Fig.
(TIFF)

S3 Fig After replacing *Q* and *K* in 2009 with their values from 2013, selection for resistance in *H*. *armigera* and *H*. *punctigera* for model L under three scenarios about crop planting: (i) cotton (Bt and conventional) is only available in generations 2–4 (black lines); (ii) like (i) but 10% of cotton is also available for generation 5 (red lines and triangles); and (iii) like (i) but 10% of cotton is also available for generation 1 (green lines and circles). This figure is identical to Fig 2 in the main text, except values of *Q* and *K* in 2009 are replaced with their values in 2013. The relative contributions of different crops and native vegetation for each of six years are based on field data from Cecil Plains. The top panels give *K*, the total available breeding habitat (Bt cotton, mandated refuges, and unmandated refuges) and the next-lower panels give *Q*, the proportion of breeding habitat that is refuge. Years are demarcated by vertical dashed lines. The six-year patterns in *Q* and *K* are repeated in the simulations, so the values in first generation (first point on the left) arise from the values in the thirtieth generation (last point on the right). The next-lower panels are the log_10_ densities of eggs in refuges, measured before density-dependent mortality. The bottom panels give the strength of selection for resistance, Ф_*t*_ (Eq 1). For all simulations, *F* = 50 and survival of diapausing pupae is 0.2. Habitat preferences are given in Table 1.
(TIFF)

S4 Fig **After replacing *Q* and *K* in 2009 with their values from 2013, relative time to control failure for *H*. *armigera* (A,B) and *H*. *punctigera* (C,D) versus the proportion of cotton crop (Bt and conventional) that is either (A,C) available late to generation 5 or (B,D) available early to generation 1.** This figure is identical to Fig 3 in the main text, except values of *Q* and *K* in 2009 are replaced with their values in 2013. Results are for model L (black lines), model A (dashed blue lines and +'s) and model I (turquoise dashed lines with x's). Time to control failure is given relative to the case of strict cotton cropping that is available to only generations 2–4, with time to control failure given when the allele frequency of resistance to Cry2Ab reaches 0.5; resistance to Cry2Ab occurs before resistance to Cry1Ac. Habitat proportions are given by the landscape at Cecil Plains for 2009/10-2013/14, and the habitat preferences for each species are the same as Fig 2.
(TIFF)

S1 File Compressed (zipped) file containing Matlab computer code and the input file used to create S1 and S2 Figs. (ZIP)

## References

[1] Benbrook CM. Impacts of genetically engineered crops on pesticide use in the U.S.–the first sixteen years. Environmental Sciences Europe. 2012;24:1–13.

[2] Huang J, Hu R, Rozelle S, Pray C. Insect-resistant GM rice in farmers' fields: Assessing productivity and health effects in China. Science. 2005;308:688–90. 10.1126/science.1108972 15860626

[3] Fitt GP. Have Bt crops led to changes in insecticide use patterns and impacted IPM? In: Romeis J, Shelton TM, Kennedy G, editors. Integration of insect resistant GM crops within IPM programs Progress in biological control series, vol 5 Heidelberg Germany: Springer-Verlag; 2008 p. 303–28.

[4] Downes S, Mahon R. Successes and challenges of managing resistance in *Helicoverpa armigera* to Bt cotton in Australia. GM crops & food. 2012;3(3):228–34. 22572906 10.4161/gmcr.20194

[5] Lu Y, Wu K, Jiang Y, Guo Y, Desneux N. Widespread adoption of Bt cotton and insecticide decrease promotes biocontrol services. Nature. 2012;487(7407):362–5. 10.1038/nature11153 22722864

[6] Storer NP, Peck SL, Gould F, Van Duyn JW, Kennedy GG. Spatial processes in the evolution of resistance in *Helicoverpa zea* (Lepidoptera: Noctuidae) to Bt transgenic corn and cotton in a mixed agroecosystem: a biology-rich stochastic simulation model. Journal of Economic Entomology. 2003;96(1):156–72. 12650359 10.1093/jee/96.1.156

[7] Peck S, Gould F, Ellner S. Spread of resistance in spatially extended regions of transgenic cotton: Implications for management of *Heliothis virescens* (Lepidoptera: Noctuidae). Journal of Economic Entomology. 1999;92:1–16.

[8] Ives AR, Glaum PR, Ziebarth NL, Andow DA. The evolution of resistance to two-toxin pyramid transgenic crops. Ecological Applications. 2011;21(2):503–15. 21563580 10.1890/09-1869.1

[9] Gould F, Tabashnik BE. Bt-cotton resistance management In: Mellon M, Rissler J, editors. Now or never: serious new plans to save a natural pest control. Cambridge, MA: Union of Concerned Scientists; 1998 p. 67–105.

[10] Gould F. Sustainability of transgenic insecticidal cultivars: Integrating pest genetics and ecology. Annual Review of Entomology. 1998;43:701–26. 10.1146/annurev.ento.43.1.701 15012402

[11] Gould F, Anderson A, Reynolds A, Bumgarner L, Moar W. Selection and genetic analysis of a *Heliothis virescens* (Lepidoptera, Noctuidae) strain with high levels of resistance to *Bacillus thuringiensis* toxins. Journal of Economic Entomology. 1995;88(6):1545–59.

[12] Tabashnik BE, Brevault T, Carriere Y. Insect resistance to Bt crops: lessons from the first billion acres. Nature Biotechnology. 2013;31(6):510–21. 10.1038/nbt.2597 23752438

[13] Gould F. Simulation models for predicting durability of insect resistant germ plasm: a deterministic model. Environmental Entomology. 1986;15(1):1–10.

[14] Comins HN. Tactics for resistance management using multiple pesticides. Agriculture Ecosystems & Environment. 1986;16(2):129–48.

[15] Tabashnik BE, Gould F, Carrière Y. Delaying evolution of insect resistance to transgenic crops by decreasing dominance and heritability. Journal of Evolutionary Biology. 2004;17(4):904–12. 10.1111/j.1420-9101.2004.00695.x 15271091

[16] Mani GS. Evolution of resistance in the presence of two insecticides. Genetics. 1985;109:761–83. 3988040 10.1093/genetics/109.4.761 PMC1202506

[17] Roush RT. Two-toxin strategies for management of insecticidal transgenic crops: can pyramiding succeed where pesticide mixtures have not? Philosophical Transactions of the Royal Society of London Series B-Biological Sciences. 1998;353(1376):1777–86.

[18] Caprio MA. Evaluating resistance management strategies for multiple toxins in the presence of external refuges. Journal of Economic Entomology. 1998;91:1021–31.

[19] Gould F. Impact of small fitness costs on pest adaptation to crop varieties with multiple toxins: A heuristic model. Journal of Economic Entomology. 2006;99(6):2091–9. 17195678 10.1603/0022-0493-99.6.2091

[20] Gahan LJ, Ma YT, Coble MLM, Gould F, Moar WJ, Heckel DG. Genetic basis of resistance to Cry1Ac and Cry2Aa in *Heliothis virescens* (Lepidoptera: Noctuidae). Journal of Economic Entomology. 2005;98(4):1357–68. 16156591 10.1603/0022-0493-98.4.1357

[21] Gould F. The evolutionary potential of crop pests. American Scientist. 1991;79:496–507.

[22] Zhao JZ, Cao J, Li YX, Collins HL, Roush RT, Earle ED, et al Transgenic plants expressing two *Bacillus thuringiensis* toxins delay insect resistance evolution. Nature Biotechnology. 2003;21(12):1493–7. 10.1038/nbt907 14608363

[23] United States Environmental Protection Agency. Biopesticides registration action document: *Bacillus thuringiensis* (Bt) plant incorporated protectants In: Office of Pesticide Programs B, and Pollution Prevention Division, editor.: Environmental Protection Agency; 2001.

[24] Matten SM, Hellmich RL, Reynolds A. Current resistance management strategies for Bt corn in the United States. Transgenic Crop Protection: Concepts and Strategies. 2004:261–88.

[25] Ives AR, Andow DA. Evolution of resistance to Bt crops: directional selection in structured environments. Ecology Letters. 2002;5:792–801.

[26] Onstad DW, Gould F. Modeling the dynamics of adaptation to transgenic maize by European corn borer (Lepidoptera: Pyralidae). Journal of Economic Entomology. 1998;91:585–93.

[27] Tabashnik BE, Croft BA. Managing pesticide resistance in crop-arthropod complexes: interactions between biological and operational factors. Environmental Entomology. 1982;11:1137–44.

[28] Caprio MA. Source-sink dynamics between transgenic and non-transgenic habitats and their role in the evolution of resistance. Journal of Economic Entomology. 2001;94:698–705. 11425026 10.1603/0022-0493-94.3.698

[29] Tabashnik BE, Gassmann AJ, Crowder DW, Carrière Y. Insect resistance to Bt crops: evidence versus theory. Nature Biotechnology. 2008;26(2):199–202. 10.1038/nbt1382 18259177

[30] Zalucki MP, Adamson D, Furlong MJ. The future of IPM: whither or wither? Aust J Entomol. 2009;48:85–96.

[31] Titmarsh IJ. Mortality of immature Lepidoptera: a case study with *Heliothis* (Lepidoptera: Noctuidae) in agricultural crops on the Darling Downs. Brisbane, Australia: University of Queensland; 1992.

[32] Matthews M. Heliothine moths of Australia. In: CSIRO, editor. Collingwood, Australia: CSIRO Publishing; 1999.

[33] Kriticos DJ, Ota N, Hutchison WD, Beddow J, Walsh T, Tay WT, et al The potential distribution of invading *Helicoverpa armigera* in north america: Is it just a matter of time? PloS One. 2015;10(3). 10.1371/journal.pone.0119618 PMC4364701 25786260

[34] Gregg PC, Fitt GP, Zalucki JM, Murray DAH. Insect migration in an arid continent. II. *Helicoverpa* spp. in eastern Australia In: Drake AV, and Gatehouse A.G, editor. Insect Migration: Tracking Resources Through Space and Time. Cambridge: Cambridge University Press; 1995 p. 151–72.

[35] Fitt GP, Dillon ML, Hamilton JG. Spatial dynamics of *Helicoverpa* populations in Australia—simulation modeling and empirical-studies of adult movement. Computers and Electronics in Agriculture. 1995;13(2):177–92.

[36] Gaston LK, Shorey HH. Sex pheromones of noctuid moths. IV. An apparatus for bioassaying the pheromones of six species. Annals of the Entomological Society of America 1964;57(6):779–80.

[37] Baker GH, Tann CR, Fitt GP. A tale of two trapping methods: *Helicoverpa* spp. (Lepidoptera, Noctuidae) in pheromone and light traps in Australian cotton production systems. Bulletin of Entomological Research. 2011;101(1):9–23. 10.1017/S0007485310000106 20504387

[38] Zalucki MP, Daglish G, Firempong S, Twine P. The biology and ecology of *Heliothis armigera* (Hübner) and *Heliothis punctigera* Wallengren (Lepidoptera, Noctuidae) in Australia—what do we know. Australian Journal of Zoology. 1986;34(6):779–814.

[39] Jallow MFA, Zalucki MP. Effects of egg load on the host-selection behaviour of *Helicoverpa armigera* (Hübner) (Lepidoptera: Noctuidae). Australian Journal of Zoology. 1998;46(3):291–9.

[40] Murray DAH. Investigation into the development and survival of *Heliothis* spp. pupae in southeast Queensland. Brisbane, Australia: The University of Queensland; 1992.

[41] Wardhaugh KG, Room PM, Greenup LR. The incidence of *Heliothis armigera* (Hübner) and *Heliothis punctigera* Wallengren (Lepidoptera, Noctuidae) on cotton and other host-plants in the Namoi Valley of New South-wales. Bulletin of Entomological Research. 1980;70:113–31.

[42] Cunningham JP, Zalucki MP. Understanding heliothine (Lepidoptera: Heliothinae) pests: What is a host plant? Journal of Economic Entomology 2014;107:881–96. 25026644 10.1603/ec14036

[43] Sequeira RV, Playford CL. Trends in *Helicoverpa* spp. (Lepidoptera: Noctuidae) abundance on commercial cotton in central Queensland: implications for pest management. Crop Protection. 2002;21(6):439–47. Pii s0261-2194(01)00126-0.

[44] Rajapakse CNK, Walter GH. Polyphagy and primary host plants: oviposition preference versus larval performance in the lepidopteran pest *Helicoverpa armigera*. Arthropod-Plant Interactions. 2007;1(1):17–26.

[45] Jallow MF, Zalucki MP. Within- and between-population variation in host-plant preference and specificity in Australian *Helicoverpa armigera* (Hübner) (Lepidoptera: Noctuidae). Australian Journal of Zoology. 1996;44:503–19.

[46] Raymond M, Heckel DG, Scott JG. Interactions between pesticide genes—model and experiment. Genetics. 1989;123(3):543–51. 2599366 10.1093/genetics/123.3.543 PMC1203826

[47] Downes S, Mahon R. Evolution, ecology and management of resistance in *Helicoverpa* spp. to Bt cotton in Australia. Journal of Invertebrate Pathology. 2012;110(3):281–6. 10.1016/j.jip.2012.04.005 22537836

[48] Monsanto Australia Limited. Resistance Management Plan for Bollgard II cotton 2014–15. 2014.

[49] ESRI. ArcGIS Desktop: Release 10.2. Redlands, CA: Environmental Systems Research Institute; 2014.

[50] Maelzer DA, Zalucki MP. Analysis of long-term light-trap data for *Helicoverpa* spp. (Lepidoptera: Noctuidae) in Australia: the effect of climate and crop host plants. Bulletin of Entomological Research. 1996;89:455–63.

[51] Castañera P, Farinós GP, Ortego F, Andow DA. Sixteen Years of *Bt* Maize in the EU Hotspot: Why Has Resistance Not Evolved? PLoS ONE. 2016;11(5):e0154200 10.1371/journal.pone.0154200 27144535 PMC4856266

[52] MathWorks I. MATLAB. 8.0 ed. Natick, MA: The MathWorks, Inc.; 2014.

[53] Murray DAH, Rynne KP. Effect of host-plant on parasitism of *Helicoverpa armigera* (Lep, Noctuidae) by *Microplitis demolitor* (Hym, Braconidae). Entomophaga. 1994;39(3–4):251–5.

[54] Franzmann BA, Hardy AT, Murray DAH, Henzell RG. Host-plant resistance and biopesticides: ingredients for successful integrated pest management (IPM) in Australian sorghum production. Australian Journal of Experimental Agriculture. 2008;48(12):1594–600.

[55] Hopper KR, Powell JE, King EG. Spatial density dependence in parasitism of *Heliothis virescens* (Lepidoptera, Noctuidae) by *Microplitis croceipes* (Hymenoptera, Braconidae) in the field. Environmental Entomology. 1991;20(1):292–302.

[56] Teakle RE, Jensen JM, Mulder JC. Susceptibility of *Heliothis armigera* (Lepidoptera, Noctuidae) on sorghum to nuclear polyhedrosis virus. Journal of Economic Entomology. 1985;78(6):1373–8.

[57] Kakimoto T, Fujisaki K, Miyatake T. Egg laying preference, larval dispersion, and cannibalism in *Helicoverpa armigera* (Lepidoptera: Noctuidae). Annals of the Entomological Society of America. 2003;96(6):793–8.

[58] Sigsgaard L, Greenstone MH, Duffield SJ. Egg cannibalism in *Helicoverpa armigera* on sorghum and pigeonpea. Biocontrol. 2002;47(2):151–65.

[59] Cotton Research Development Corporation. CRDC Annual Report 2013–2014. 2014.

