# Supplementary figures and images for: Spatio-Temporal Variation in Landscape Composition May Speed Resistance Evolution of Pests to Bt Crops
Source: PLoS One. 2017 Jan 3;12(1):e0169167. doi: 10.1371/journal.pone.0169167 (PMC5207666; doi:10.1371/journal.pone.0169167)

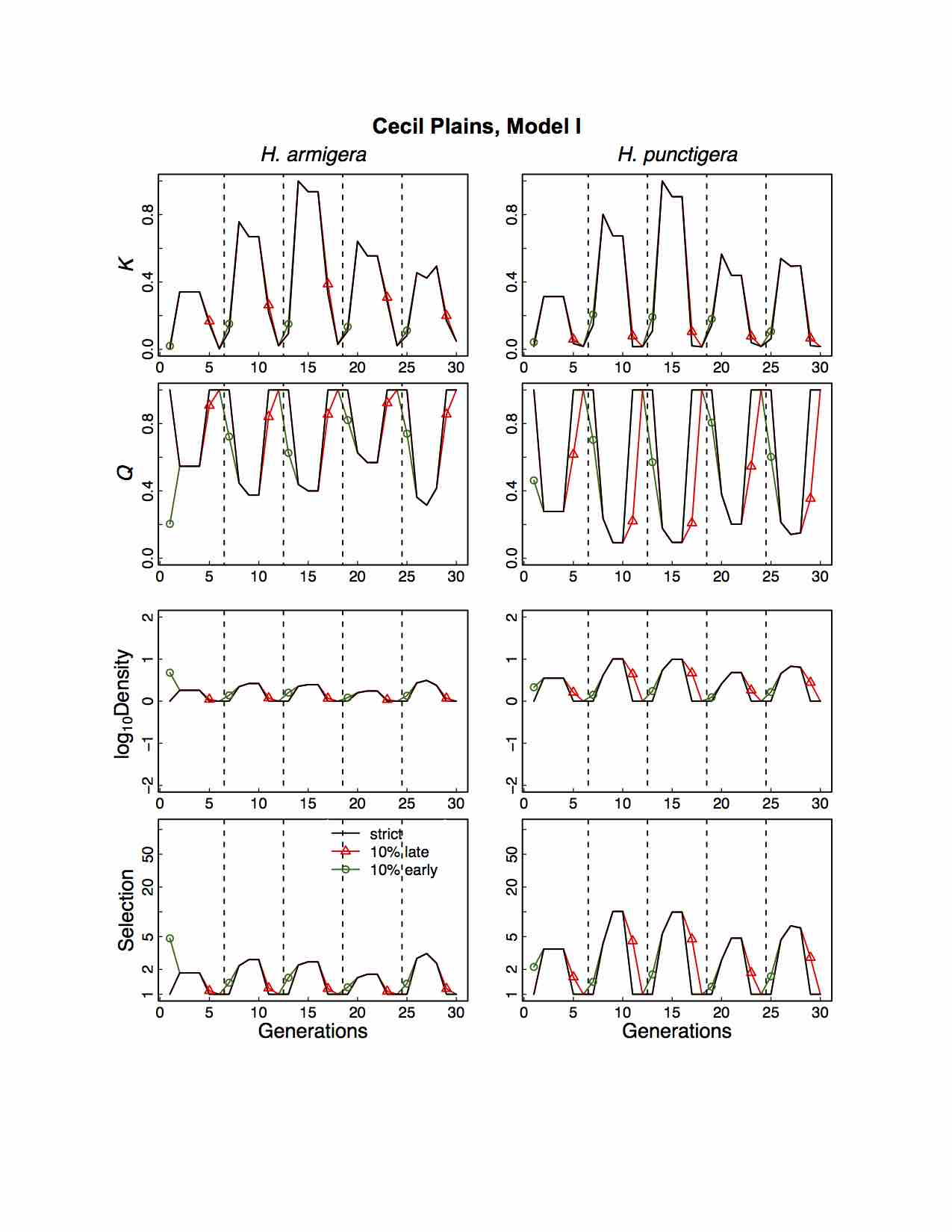

Supplement: S1 Fig — See Fig 2 in the main text. The relative contributions of different crops and native vegetation for each of six years are based on field data from Cecil Plains, Pampas, and Nandi study areas. The top panels give K, the total available breeding habitat (Bt cotton, mandated refuges, and unmandated refuges) and the next-lower panels give Q, the proportion of breeding habitat that is refuge. Years are demarcated by vertical dashed lines. The 6-year patterns in Q and K are repeated in the simulations, so the values in first generation (first point on the left) arise from the values in the thirtieth generation (last point on the right). The next-lower panels are the log10 densities of larvae in refuges, measured before density-dependent mortality. The bottom panels give the strength of selection for resistance, Фt (Eq 1). For all simulations, F = 50 and survival of diapausing pupae is 0.2. Habitat preferences are given in Table 1. (TIFF) [file pone.0169167.s001.tiff]

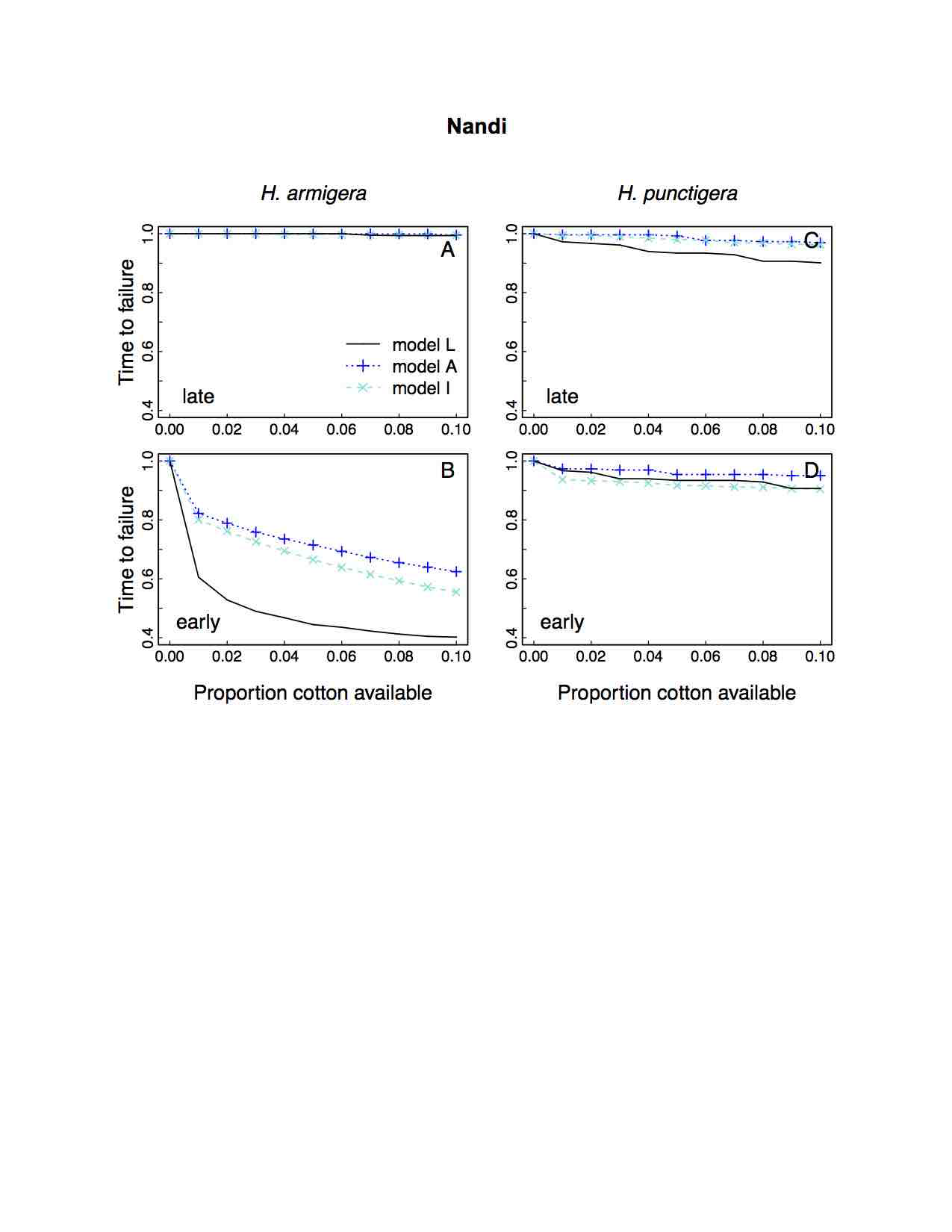

Supplement: S2 Fig — For the Cecil Plains, Pampas, and Nandi landscapes, the relative time to control failure for H. armigera (A,B) and H. punctigera (C,D) versus the proportion of cotton crop (Bt and conventional) that is either (A,C) available late to generation 5 or (B,D) available early to generation 1. See Fig 3 in the main text. Results are for model L (black lines), model A (dashed blue lines and +'s) and model I (turquoise dashed lines with x's). Time to control failure is given relative to the case of strict cotton cropping that is available to only generations 2–4, with time to control failure given when the allele frequency of resistance to Cry2Ab reaches 0.5; resistance to Cry2Ab occurs before resistance to Cry1Ac. Habitat proportions are given by each landscape 2009/10-2013/14, and the habitat preferences for each species are the same as S1 Fig. (TIFF) [file pone.0169167.s002.tiff]

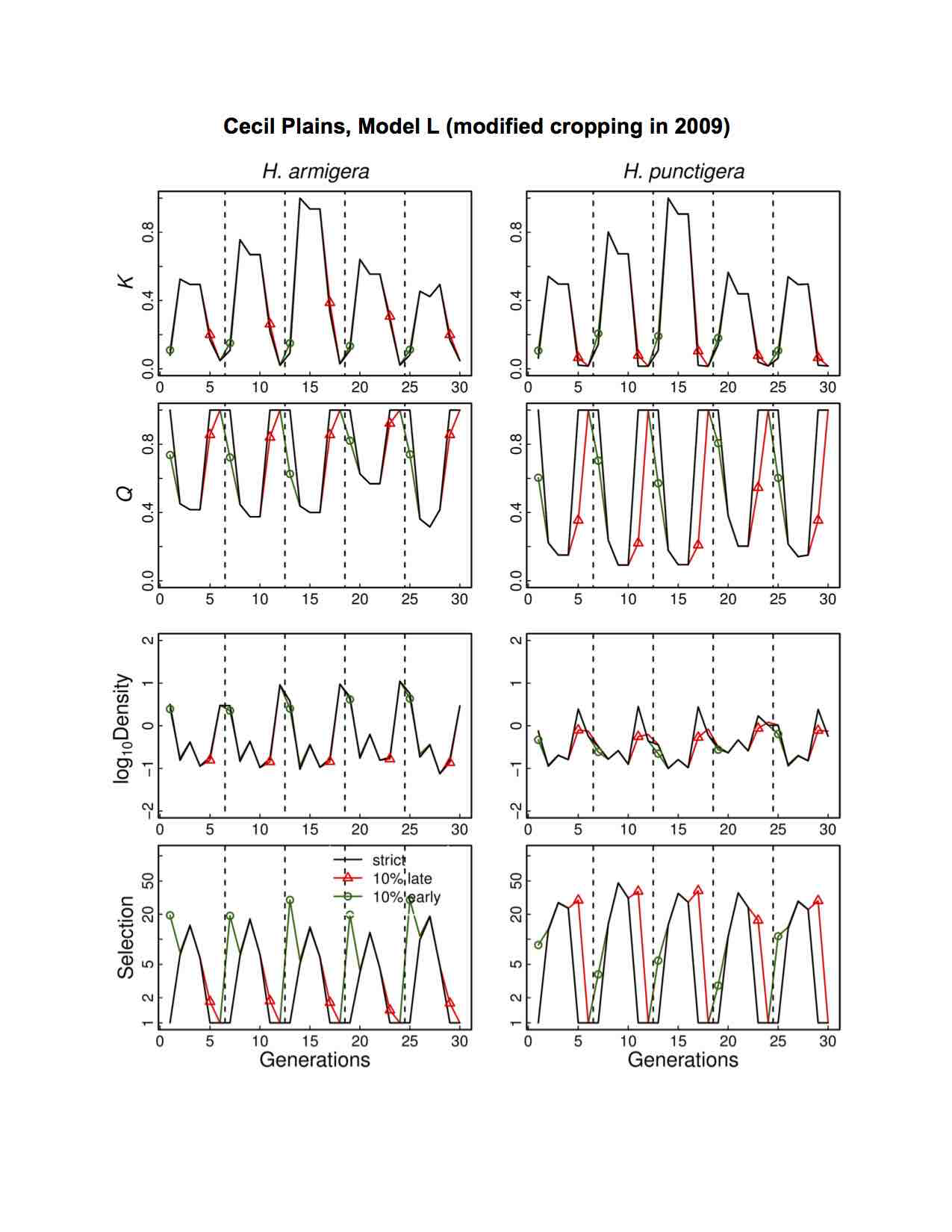

Supplement: S3 Fig — This figure is identical to Fig 2 in the main text, except values of Q and K in 2009 are replaced with their values in 2013. The relative contributions of different crops and native vegetation for each of six years are based on field data from Cecil Plains. The top panels give K, the total available breeding habitat (Bt cotton, mandated refuges, and unmandated refuges) and the next-lower panels give Q, the proportion of breeding habitat that is refuge. Years are demarcated by vertical dashed lines. The six-year patterns in Q and K are repeated in the simulations, so the values in first generation (first point on the left) arise from the values in the thirtieth generation (last point on the right). The next-lower panels are the log10 densities of eggs in refuges, measured before density-dependent mortality. The bottom panels give the strength of selection for resistance, Фt (Eq 1). For all simulations, F = 50 and survival of diapausing pupae is 0.2. Habitat preferences are given in Table 1. (TIFF) [file pone.0169167.s003.tiff]

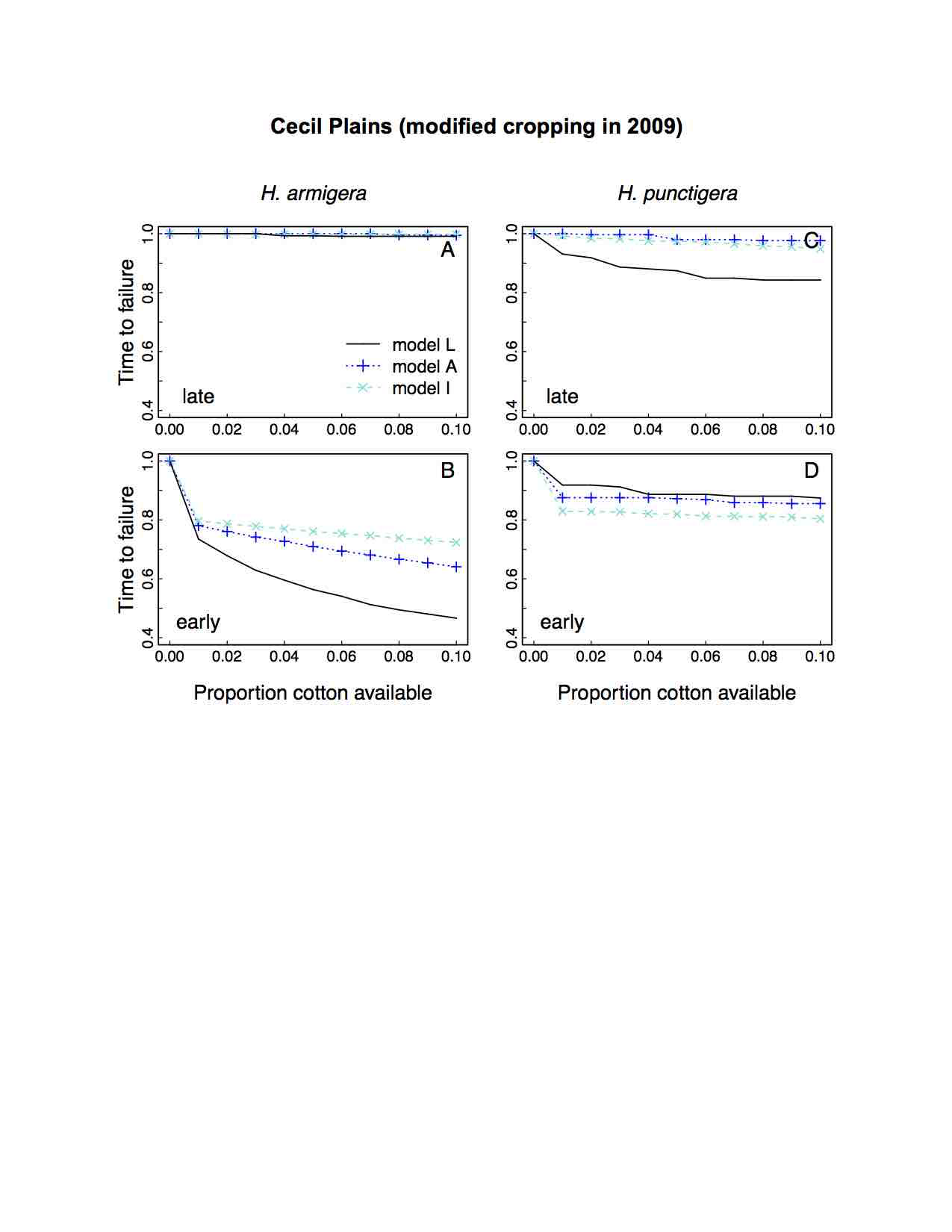

Supplement: S4 Fig — After replacing Q and K in 2009 with their values from 2013, relative time to control failure for H. armigera (A,B) and H. punctigera (C,D) versus the proportion of cotton crop (Bt and conventional) that is either (A,C) available late to generation 5 or (B,D) available early to generation 1. This figure is identical to Fig 3 in the main text, except values of Q and K in 2009 are replaced with their values in 2013. Results are for model L (black lines), model A (dashed blue lines and +'s) and model I (turquoise dashed lines with x's). Time to control failure is given relative to the case of strict cotton cropping that is available to only generations 2–4, with time to control failure given when the allele frequency of resistance to Cry2Ab reaches 0.5; resistance to Cry2Ab occurs before resistance to Cry1Ac. Habitat proportions are given by the landscape at Cecil Plains for 2009/10-2013/14, and the habitat preferences for each species are the same as Fig 2. (TIFF) [file pone.0169167.s004.tiff]
